# Historical Perspectives on Ethical and Regulatory Aspects of Human Participants Research: Implications for Oncology Clinical Trials in Africa

**DOI:** 10.1200/JGO.19.00196

**Published:** 2020-07-02

**Authors:** Bodour Salhia, Victoria Olaiya

**Affiliations:** ^1^ Department of Translational Research, University of Southern California, Norris Comprehensive Cancer Center, Keck School of Medicine, Los Angeles, CA; ^2^ Synteract Limited, Cambridgeshire, United Kingdom

## Abstract

Clinical trials research involving human participants has led to numerous medical advances. Historically, however, clinical trials research was the source of major concerns for the safety and welfare of the human participants taking part in these studies. The ethical principles of autonomy, beneficence, and justice came about in response to medical atrocities, and regulations were ultimately put in place to protect the rights and welfare of human participants and to maintain the public trust in the research enterprise. Today, clinical trials are one of the most heavily regulated practices in the world, and yet still not all people are provided the same oversights and protections, with improprieties disproportionately affecting poor-resource nations and vulnerable populations. As Africa approaches the post–communicable disease era, cancer is set to take the lead as the most burdensome disease, making the need for oncology clinical trials in Africa greater than ever before. Africa represents a heterogeneous market with 55 countries, most with their own National Regulatory Agency (NRA) and each with varying levels of regulatory maturity. This diversity creates a highly complex regulatory environment and causes challenges when bringing drugs to market. There is a large need for harmonization and increased collaboration between the African nations’ NRAs. In addition, many African countries need to be better equipped to handle research ethics committees and/or learn how to rely on neighboring countries with more established ethics committees. Well-run clinical trials offer solutions to national health care problems, and all people deserve equal access to their benefits.

## INTRODUCTION

Through the centuries, human participants research has led to numerous breakthroughs in medicine, which were often accelerated with technologic advances. However, concerns about the ethics of research involving human participants have a long history. In the past half century, the level of oversight on human participants research has exploded from almost none to what is now an exhaustive system of protections. Still, these oversights and protections are not uniform throughout the world, and many nations, especially poor-resource nations, remain at risk for ethical improprieties. The Pure Food and Drug Act was passed in 1906 in the United States. This was the first of a series of protections for consumers before which there were no governmental or institutional regulations governing the ethical use of human participants research.^1^ As such, the US Food and Drug Administration (FDA), the Common Rule, and the institutional review board (IRB) did not yet exist.^1^ Research involving human participants was fraught with danger and suffering. Still, the concepts of the importance of justice and morality have ancient roots. The ancient Egyptians believed in Ma’at, which refers to their notions of truth, balance, order, harmony, law, morality, and justice.^2,2a^ Ma’at was also the goddess who manifested these concepts.^2,2a^ The ancient Hippocratic Oath states that it is a physician’s duty to avoid harming the patient, but the oath was not subscribed to by the majority of doctors until the late postmodern era. Ultimately, measures for the protection of human participants have mostly come in response (albeit a late response) to reported abuses and scandals. Society has endured significant harm as a result of research misconduct that lacked in basic concepts of morality, justice, law, and balance. As a result, our society has learned how to ensure the ethical conduct of research while continuing in the pursuit of scientific advancement for the benefit of humanity. The ethical principles and regulations that have been developed over the years were designed to protect the rights and welfare of human participants in research and maintain the public trust in the research enterprise.

**Context**

**Key Objective**
What were the circumstances that led to modern-day research ethics boards? Do patients with cancer in Africa have access to clinical trials? What are the existing oversights to protect the welfare of clinical trial participants in countries with limited resources?
**Knowledge Generated**
The ethical principles of autonomy, beneficence, and justice evolved in response to medical abuses on human participants. Regulations were put in place to protect the rights and welfare of human participants and are governed by research ethics boards; as a result, clinical trials are now heavily regulated. Approximately 10% of clinical trials occur in developing countries. Unlike in the United States or European Union, sub-Saharan Africa has a diverse and complex regulatory environment. This presents challenges that hinder oversights and protections and lead to improprieties that disproportionately affect poor-resource nations in Africa and vulnerable populations.
**Relevance**
Well-run clinical trials provide solutions to national health care problems. There is a need to continue to develop clinical trial best practices across Africa. Everyone deserves equal access to clinical trials, and we must strive to achieve that access.

## DEFINITIONS OF HUMAN PARTICIPANTS RESEARCH AND CLINICAL TRIALS

In essence, human participant research, as defined by the National Institutes of Health (NIH) of the Department of Health and Human Services (DHHS) is “systematic, scientific investigation that can be either interventional (a ‘trial’) or observational (no ‘test article’) and involves human beings as research subjects.”^16^ According to the federal policy known as the Common Rule, as defined in 45 CFR 46, a human participant is “a living individual about whom an investigator (whether professional or student) conducting research obtains information or biospecimens through intervention or interaction with the individual, and uses, studies, or analyzes the information or biospecimens; or obtains, uses, studies, analyzes, or generates identifiable private information or identifiable biospecimens.”^3^

Specifically, clinical trials are wide ranging in their scope and include prevention, screening, diagnostic, treatment, behavioral, and quality-of-life trials. According to the NIH, a clinical trial is a “research study in which one or more human subjects are prospectively assigned to one or more interventions (which may include placebo or other control) to evaluate the effects of those interventions on health-related biomedical or behavioral outcomes.”^4^ Prospective assignment refers to a predefined process specified in an approved protocol that stipulates the assignment of research participants to one or more arms of a clinical trial, and intervention is defined as a manipulation of the participant’s environment for the purpose of modifying one or more health-related biomedical or behavioral processes and/or end points.^4^ The last component, a “health-related biomedical or behavioral outcome,” is defined as the prespecified goal(s) or condition(s) that reflect the effect of one or more interventions on human participants’ biomedical or behavioral status or quality of life.^4^ All 3 conditions must be met for the research study to be considered a clinical trial by the NIH, and therefore, all human participants research does not fall under the clinical trials category. For example, collecting samples from patients either at a single time point or over time without intervention or the measuring of an outcome would qualify as an observational study and not as a clinical trial. One can use the decision tool on the NIH Web site to determine whether the human participants research meets the NIH definition of a clinical trial.^5^

## HISTORICAL ACCOUNTS OF HUMAN EXPLOITATION AND ABUSES AND THE REGULATIONS THAT FOLLOWED

In 1966, Beecher^6^ published the article “Ethics and Clinical Research” in *The New England Journal of Medicine*. Beecher, a senior member of the anesthesiology faculty at Harvard Medical School, examined the ethical practices of 22 studies conducted by respected investigators and published in prestigious medical journals.^6,7^ He discovered in each of these studies ethical improprieties, including but not limited to lack of informed consent and increased risk to human participants.^7^ Among the most horrific examples of ethical abuses in our modern history is the case of the Tuskegee Study of Untreated Syphilis in the Negro Male (commonly known as the Tuskegee Syphilis Study).^7-9^ This was a medical research project conducted by the US Department of Health from 1932 to 1972 that examined the natural course of untreated syphilis in 400 African American men. According to the Centers for Disease Control and Prevention, the participants, all impoverished sharecroppers from Macon County, Alabama, did not know they were being studied and were not told that they had syphilis. Researchers withheld treatment even when it became available in the late 1940s with the availability of penicillin. Most participants who attended the Tuskegee clinic thought they were getting treatment for “bad blood.” The study concluded in 1972, and when stories about the study leaked to the public, it caused an outcry. This and other cases of abuse, including extensive medical research conducted on prisoners in correction facilities; the Willowbrook studies (1956-1970), in which children with intellectual disabilities were deliberately infected with the hepatitis virus^7,10^; and the Jewish Chronic Disease Hospital Study (1963), in which live cancer cells were injected into 22 cognitively impaired patients, contributed to the public’s concern over medical research.^7,10^

However, laws regarding the ethics of research involving human participants first developed as the result of the Nazi regime’s atrocities during World War II.^7^ On November 19, 1945, the International Military Tribunal was decreed by the Allied powers. This tribunal was a series of trials held against major war criminals and Nazi sympathizers. The Doctors’ Trial of 1947 became the first trial conducted under the Nuremberg Military Tribunals. In this trial, 23 physicians from the Nazi Party were tried for atrocious experiments they carried out on helpless and unwilling prisoners of war and for crimes against humanity. Many of the unfathomable medical experiments took place at the Auschwitz concentration camp, where demeaning numbers were tattooed onto the arms of prisoners to identify their bodies after death.^7,11^

As part of the verdict, the court ordered rules for “Permissible Medical Experiments,” known as the Nuremberg Code, published in 1947. The 3 basic elements of the Nuremberg Code state that research requires voluntary informed consent, that the benefits of meritorious research outweigh the risks, and that the participants have the right and ability to terminate participation at any time without consequences to the quality of their care. The Nuremberg Code became the foundation for subsequent ethical codes and research regulations. In 1964, the World Medical Association released the Declaration of Helsinki, which built on the principles of the Nuremberg Code.^12^ The trials also led to the Council for International Organizations of Medical Sciences (CIOMS), which was established jointly by the WHO and the United Nations Educational, Scientific, and Cultural Organization in 1949 as an international, nongovernmental, nonprofit organization with the mission to advance public health through guidance on health research, including ethics, medical product development, and safety. The development of the CIOMS Guidelines, known as the International Ethics Guidelines for Biomedical Research Involving Human Subjects of 1982, has especially influenced research ethics in poor-resource nations such as those in Africa.^13^

Astonishingly, the research improprieties in the United States and abroad did not prompt US congressional deliberations about human participants research oversight until 1974, which came even after the adoption of the Animal Welfare Act in 1966 (intended for the protection of research animals). Congress’s first legislation to protect the rights and welfare of human participants was the National Research Act of 1974. This led to the creation of the National Commission for Protection of Human Subjects of Biomedical and Behavioral Research, which issued the Belmont Report in 1976 and led to the modern-day IRB.^7,14,15^ Autonomy, beneficence, and justice provide the framework of the Belmont Report and provide the bioethical precepts of conducting human participants research. The Belmont Report identified the following 3 basic principles relevant to the ethical conduct of research involving human participants: respect for persons—all individuals should be treated as autonomous agents, and persons with diminished autonomy are entitled to protection; beneficence—researchers should maximize possible benefits and minimize possible harm; and justice—all persons should be treated equally, and the selection of research participants should be scrutinized so that no one is systematically selected on the basis of race, ethnicity, class, or other factors.^15,16^

Although the Belmont Report emphasizes individual autonomy, it also recognizes that when individuals who belong to vulnerable populations are involved in research, harm may come not necessarily to the individual participants but to the group as a whole, including those who did not actually take part in the research. Most formal policies and regulations on the protection of human participants research include this risk as an adjunct to the Belmont Report’s 3 core principles and attribute group harms to the misuse and misinterpretation of research findings. Groups at increased risk include minority populations, such as African Americans, Hispanics, American Indians, and other racial and ethnic minorities both in the United States and abroad that have historically faced racism and oppression; groups that are socioeconomically disadvantaged and have less access to education, social services, and health care; and groups with behaviors outside of societal norms such as sex workers and drug users. Similarly, conditions such as HIV/AIDS, leprosy, lung cancer, epilepsy, or schizophrenia, which lead to stigma, make research operationally and ethically challenging.^17^ Research findings causing group harm or stigma can lead to genetic determinism and economic, political, social, educational, and cultural harms. These can be difficult to measure, predict, and reverse. Conditions that are still stigmatized and vulnerable populations need to be studied to develop interventions to reduce stigma and harm to these groups. A report by Millum et al^17^ concluded that overprotecting vulnerable populations, whether by excluding them altogether, by instituting excessive protections, or by refusing to engage with controversial questions, should be avoided. Rather, investigators and ethics committees need to be more attentive to the problems and associated risks.

Therefore, research oversight capacity is critical for the protection of human research participants, as well as to prevent exploitation of populations, communities, institutions, and countries. The IRB, also known as an independent ethics committee, ethical review board, or research ethics board, or research ethics committee (REC) outside of the United States, is an administrative body established to protect the rights and welfare of human research participants recruited to participate in research activities conducted under the auspices of the institution with which it is affiliated. The IRB, as with other ethics committees, is required to review all biomedical and behavioral research (whether funded or not) involving human participants before study initiation. The IRB has the authority to approve, disapprove, monitor, and require modifications of all research activities. In 1991, 16 federal agencies also formally adopted the core of the Belmont Report’s regulations in a common Federal Policy for the Protection of Human Subjects. More commonly known as the Common Rule, this set of ethics involving the protection of human participants is codified under Title 45, Part 46 of the Federal Regulations. These DHHS regulations are divided into 4 subparts and were revised to modernize and strengthen the Common Rule in July of 2015^14,16^:

- Subpart A: The Federal Policy, or Common Rule
- Subpart B: Additional protections for pregnant women, human fetuses, and neonates
- Subpart C: Additional protections for prisoners
- Subpart D: Additional protections for children

The non-Western world has certainly not been immune to research and ethics abuses, with numerous reports documenting unethical research and clinical trials in Africa and elsewhere. The 1990s were especially trying times for medical research in Africa. For example, a British anesthetist by the name of Dr Richard Gladwell McGown, working in Zimbabwe, was charged with murder for the death of 6 patients.^18,19^ He conducted a study on 500 mostly black patients to test new drugs and anesthetics without the approval of the National Drug Authority. In 1996, Pfizer (New York, NY) sent a team of US doctors to Kano, Nigeria, to compare trovafloxacin (Trovan), an experimental drug to treat bacterial meningitis, with ceftriaxone.^17,20^ Eleven children died, and others endured brain damage in the control arm allegedly as a result of receiving a suboptimal dose of ceftriaxone. Pfizer was sued through the US courts and settled out of court. In addition to these and many other documented reports of research abuses in Africa and poor-resource nations in other parts of the world, the majority of incidences related to breaches in ethical conduct most likely go unreported. This may be a result of weak regulatory systems and mostly litigation-free environments. There is also a tendency to underreport adverse events, and volunteer participants are generally happy to participate in what they regard as innovative medical breakthrough research and treatment.^19^

## CULTURAL AND INTERNATIONAL ETHICAL CONSIDERATIONS

Autonomy, beneficence, and justice provide the framework of the Belmont Report and provide the bioethical precepts of conducting human participants research in the United States and other Western countries. Interpreting and applying these principles in another cultural setting may be challenging. Controversies have erupted concerning the ethics of biomedical research sponsored by wealthy nations and conducted in resource-poor countries. In developing countries characterized by such factors as poverty, low literacy levels, and limited access to health care, informed consent becomes especially complex. Often there is a language and/or cultural barrier between the researcher and the participant. Beliefs about disease causation may also differ. Cultural perceptions of individuality also influence the decision-making process. In Western cultures, decisions are made independently. However, in an African context, decision making is often communitarian and semiotic, involving consultations with family and/or community members where issues at stake are discussed in a forum (imbizo).^21^ The elders of the community usually preside at this forum, and all viewpoints are shared.^19,21^ For meaningful informed consent, researchers have to appreciate and respect the cultures and beliefs of the groups they recruit for research studies.^19^ Questions have also been raised about whether participants in vulnerable situations truly consented or whether they were paid to participate or misled to believe that the new treatment is a medical breakthrough and will cure them.

Clinical trials in poor-resource nations have also generated considerable debate about whether the use of a placebo (eg, zidovudine placebo trials in Africa) is ethically just, especially when there is a course of effective treatment used elsewhere in the world but that is unavailable in the countries in which the study was conducted.^22-24^ Another important consideration to the implementation of the Belmont Report in low-resource nations is the distribution of benefits and burdens inherent in the research design. For example, it was always the intent of the manufacturer to market the drug lucinactant in the United States and other developed countries.^25^ Thus, the benefits would go to infants in the United States and perhaps other more developed countries, while the burdens (risks) of the research would be borne by infants in Latin America, where the study was conducted but the drug unaffordable by its population. This distinction between the population asked to test the drug and the population in which the drug would be marketed violates the principle of distributive justice (ie, an unequal distribution of benefit and burden). Redesigning the study might have improved its ethical foundation. For example, the drug might have been provided to the Latin American country free for a period of time or at a discounted price.^26^ Thus, it is essential that local ethics committees have the knowledge, expertise, competence, and authority to review and both approve and reject, when appropriate, clinical trials.

For these reasons, US regulations mandate a study to be reviewed and approved by an IRB in both the United States and the host country. US regulations also require that researchers adhere to the national guidelines or regulations of the host country.^19^ Countries that do not have review boards have the option to request assistance from neighboring countries. For example, in Guinea-Bissau, once a research proposal is disclosed to the government and deemed appropriate, it can be sent to the National Ethics Committee in The Gambia for review and approval.^19^ It is critical for researchers to have a clear understanding of relevant cultural issues of host countries, especially those of developing nations, to conduct studies that truly embody the essence of autonomy, beneficence, and justice and are free of group harms.The Regional Committee for Africa of the WHO conducted a survey study to determine the existence and utility of RECs across Africa.^27,28^ The study findings were that 36% of member countries had no RECs. In the countries that did have RECs, most RECs met monthly, 5 met quarterly, and 1 never met. Milford et al^29^ studied the resource needs of African RECs regarding HIV vaccine trial preparedness and found that 97% of African RECs felt they did not have adequate training in ethics and HIV vaccine trials and 80% felt they did not have adequate training in health research ethics.^27,28^ Hyder et al^30^ reported similar findings, while also demonstrating that the majority of researchers were middle-aged males who were physicians used by academic institutions, carrying out research on a part-time basis. The study by Hyder et al^30^ also indicated the need for local language consent forms and approval letters. Concerns for confidentiality protection of participants were raised by US IRBs in significantly higher proportions than by host country IRBs. In 2007, the Bill and Melinda Gates Foundation supported the African Malaria Network Trust to conduct a broad survey of 31 RECs in Africa to identify institutional needs.^19^ The results of the survey demonstrated that committees lacked guidelines and standard operating procedures, training, and electronic data management and archiving systems. Fast forwarding to 2019, many issues and inefficiencies such as obtaining timely approvals still exist in poor-resource nations, risking the ethical and data integrity of clinical trials.

## REGULATORY ASPECTS OF ONCOLOGY CLINICAL TRIALS

Before a clinical trial can begin, it needs to be reviewed and authorized by a regulatory agency to ensure that human participants are protected and to provide assurance that the data generated are accurate and credible. In the United States, an Investigational New Drug application must be filed with the FDA to obtain permission to start human clinical trials. In Europe, a Clinical Trial Application is filed with the European Medicines Agency. Detailed documentation about the Investigational Medicinal Product, which should include preclinical data, safety evaluation, manufacturing process, toxicology, and safety pharmacology, is required to be submitted as part of regulatory documents for obtaining clinical trial authorization. Unlike the United States or European Union, sub-Saharan Africa is not a single unified market. Rather, it is made up of 46 different markets each with its own National Regulatory Agency (NRA) and each having varying levels of regulatory maturity. This diverse regulatory environment leads to challenges when bringing a drug to market. In June 2016, during a meeting of the African Vaccine Regulatory Forum, heads of NRAs and national ethics committees across Africa agreed to harmonize their practices to strengthen regulatory oversight of clinical trials across the continent.^31^ With disease burden on the rise in Africa and disproportionately affecting the developing world at large, 10% of the nearly 100,000 clinical trials conducted annually worldwide are occurring in developing countries.^19,32^ Well-run clinical trials can provide national health care solutions to national problems, build local health care capacity, provide employment and access to new health care services and treatments,^19^ improve the quality of care, and build confidence in health care practices.

Clinical trials are usually carried out in different stages, called phases. In the preclinical stage of anticancer drugs, there are specific nonclinical guidelines that must be adhered to and that regulatory authorities will look out for when the regulatory dossier is submitted for authorization. There are 4 main phases of clinical trials—phases I to IV—although some trials may be categorized as phase 0 trials. Phase 0 trials involve a small number of patients with cancer (approximately 10-20 patients) and test a small drug dose. These trials aim to determine whether the study drug targets the cancer cells and whether there are early signs of efficacy. Phase I trials enroll 20-80 patients. The main aim of phase I clinical trials is to determine the safety of a new drug and the maximum-tolerated dose. If a drug is proven to be safe, it advances to phase II. Phase II trials enroll 100-300 patients and aim to determine therapeutic efficacy as well as to continue to monitor drug safety and the adverse effect profile. Phase III trials enroll hundreds of patients and aim to compare the new treatment to standard-of-care treatment, which serves as the control arm. Safety continues to be monitored. Patients are randomly assigned to a treatment arm to minimize bias, and the study is double-blinded, meaning neither the patient nor the doctor knows which treatment has been assigned to the participant until the study is over. Phase IV trials test a drug after it has been approved. They are conducted in thousands of patients, and the goals is to learn more about the drug’s short-lived and long-lasting adverse effects, long-term benefits, and cost-benefit analysis.^33^ There are differing clinical trial authorization time lines depending on the phase of the clinical trial. For instance, first-in-human studies tend to have shorter review and approval time lines than phase II and III studies. During the development of anticancer drugs, clinical trials usually involve patients with cancer whose disease condition is progressive and fatal. In addition, the dose levels in these clinical trials are often close to the adverse effect dose levels.

In conclusion, the purpose of ethics and regulations is to protect the rights and welfare of patients, ensuring autonomy, beneficence, and fairness. However, all people are not provided the same oversights and protections, with improprieties disproportionately affecting poor-resource nations and vulnerable populations. As we approach the post–communicable disease era in Africa, cancer will take the lead as the most burdensome ailment on the continent. As a result, the need for oncology clinical trials in Africa is greater than ever before. However, this must be tackled in concert with improving ethical standards, compliance, and reporting. In addition, many African countries need to be better equipped to handle RECs or learn how to rely on neighboring countries with more established ethics committees. Additional information on how African RECs function, including their staffing, operating procedures, strengths, and challenges, would be useful for African and international researchers working within Africa and for growing efforts to enhance ethics capacity across the continent.^28^ Researchers need to enhance their cultural competency to adjust their mindset to the specific needs of the patients who are participants in their research and also need to gain a clear understanding of the ethical guidelines of both the sponsoring and host countries. Finally, the scientific community needs to continue to debate best practices in low-resource nations. After all, everyone deserves equal access to clinical trials, and we must strive to achieve that.
